# Bioinformatic Analysis of the Nicotinamide Binding Site in Poly(ADP-Ribose) Polymerase Family Proteins

**DOI:** 10.3390/cancers13061201

**Published:** 2021-03-10

**Authors:** Garri Manasaryan, Dmitry Suplatov, Sergey Pushkarev, Viktor Drobot, Alexander Kuimov, Vytas Švedas, Dmitry Nilov

**Affiliations:** 1 Faculty of Medicine, Lomonosov Moscow State University, Lomonosov Ave. 27, bldg. 1, 119991 Moscow, Russia; garrim1@mail.ru; 2 Belozersky Institute of Physicochemical Biology, Lomonosov Moscow State University, Lenin Hills 1, bldg. 40, 119991 Moscow, Russia; d.a.suplatov@belozersky.msu.ru (D.S.); linux776@gmail.com (V.D.); kuimov@genebee.msu.su (A.K.); vytas@belozersky.msu.ru (V.Š.); 3 Faculty of Bioengineering and Bioinformatics, Lomonosov Moscow State University, Lenin Hills 1, bldg. 73, 119991 Moscow, Russia; spush.bio@gmail.com

**Keywords:** poly(ADP-ribose) polymerase, inhibitor, nicotinamide, 7-methylguanine, D-loop, tankyrase, molecular dynamics, homology modeling

## Abstract

**Simple Summary:**

The PARP family consists of 17 proteins, and some of them are responsible for cancer cells’ viability. Much attention is therefore given to the search for chemical compounds with the ability to suppress distinct PARP family members (for example, PARP-5a and 5b). Here, we present the results of a family-wide bioinformatic analysis of an important functional region in the PARP structure and describe factors that can guide the design of highly selective compounds.

**Abstract:**

The PARP family consists of 17 members with diverse functions, including those related to cancer cells’ viability. Several PARP inhibitors are of great interest as innovative anticancer drugs, but they have low selectivity towards distinct PARP family members and exert serious adverse effects. We describe a family-wide study of the nicotinamide (NA) binding site, an important functional region in the PARP structure, using comparative bioinformatic analysis and molecular modeling. Mutations in the NA site and D-loop mobility around the NA site were identified as factors that can guide the design of selective PARP inhibitors. Our findings are of particular importance for the development of novel tankyrase (PARPs 5a and 5b) inhibitors for cancer therapy.

## 1. Introduction

Poly(ADP-Ribose) polymerase proteins (PARPs 1–16) catalyze the transfer of ADP-ribose from the nicotinamide adenine dinucleotide (NAD^+^) substrate to target proteins and are involved in many cellular functions (Table 1) [1,2,3,4,5,6,7]. In particular, the activity of the founding family member PARP-1 at DNA damaged sites recruits the base excision repair proteins XRCC1, DNA polymerase β, and DNA ligase III [8,9,10]. The most studied PARP family members are PARP-1 and 2 involved in DNA repair and PARP-5a and 5b (also known as tankyrases 1 and 2) regulating the Wnt signaling pathway. In the past years, aberrant Wnt signaling was found to be implicated in numerous malignancies, including gastrointestinal cancers, leukemia, and breast cancer [11,12].

Much attention is being paid to the search for inhibitors of distinct PARP family members because of their role in breast/ovarian cancers [29,30,31,32,33], gastrointestinal cancers, and many other, non-oncological diseases [34,35,36]. A number of PARP inhibitors contain an amide group attached to an aromatic ring or lactam group built in an aromatic ring system and mimic the nicotinamide (NA) moiety of NAD^+^. They suppress enzyme activity at nanomolar concentrations but lack sufficient selectivity [37,38,39], for example, isoquinolinone derivatives [40] are able to bind to PARPs 1, 3, and 5a. FDA-approved inhibitors of PARP-1/2 (olaparib, rucaparib, and niraparib) [41,42] can cause serious side effects likely related to the nonselective interaction with numerous NAD^+^-binding proteins, including other PARP family members [43,44,45,46].

The design of highly effective inhibitors is usually focused on a region of the PARP active site responsible for binding the NA group of NAD^+^. The architecture of the NA site provides strong interaction between the substrate/inhibitor amide group and Gly863 residue (PARP-1 numbering) via two hydrogen bonds [47,48,49,50,51]. In many crystal structures, PARP inhibitors form additional interactions with the other NA site residues: A hydrogen bond with Ser904, hydrophobic contact with Ala898, and *π*-stacking with Tyr907 [39,52,53]. The above residues are crucial for the effective binding in the PARP active site, and their mutations may modulate the affinity for inhibitors. Interactions with flexible loops around the NA site may also be important for molecular recognition. In this article, we present the results of a detailed family-wide analysis of the NA binding site in PARPs 1–16 that opens up new prospects for selective PARP inhibition.

## 2. Results

### 2.1. Multiple Sequence Alignment of PARPs 1–16

**Figure 1:**
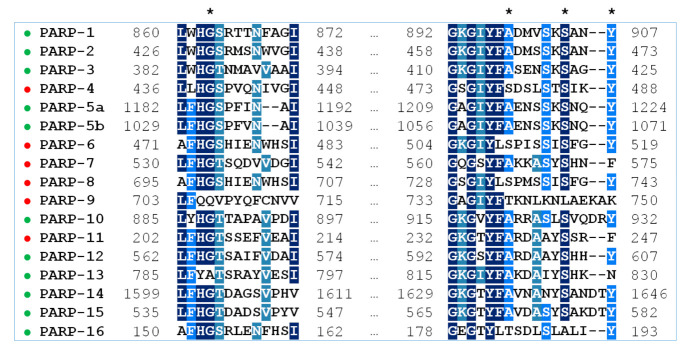
Multiple sequence alignment of PARPs 1–16 with Clustal Omega. The residues 863, 898, 904, and 907 of the NA binding site (PARP-1 numbering) are marked with an asterisk. PARP family members with known structures are marked in green, and PARPs with unknown structures in red.

A multiple sequence alignment highlighting residues of the NA binding site in PARPs 1–16 is shown in Figure 1. Gly is the most prevalent residue at position 863, Ala at position 898, Ser at position 904, and Tyr at position 907 (PARP-1 numbering). Substitutions at these positions, marked in Table 2, may have a direct impact on the substrate and inhibitor binding. Notably, the key active site residue Gly863 is substituted only in catalytically inactive PARP family members, PARP-9 and PARP-13. Crystal structures of PARPs 1–3, 5a, 5b, 10, and 12–16 are available, which helps considerably in understanding how substitutions affect the binding site architecture, and the models of PARPs 4, 6–9, and 11 can be constructed from their sequences and structures of close homologs. In Section 2.2 and Section 2.3, interactions of PARPs mediated by amino acid residues at positions 863, 898, 904, and 907 in the multiple alignment are analyzed in detail using molecular dynamics (MD) and homology modeling.

### 2.2. Modeling of PARPs with Known Structures

To model the NA binding site of PARPs 1–3, 5a, 5b, 10, and 12–16, we have selected representative crystal structures for each family member. Available structures listed in Table S1 were manually clustered into groups based on the similarity of the NA site conformation (outliers/minor conformations were excluded), and then a representative structure with the best resolution was selected from each cluster (Figure S1, Table S1). Single clusters were produced for all PARPs except PARP-5a, whose structures were divided into groups I and II differing in the D-loop conformation. The mobile D-loop of PARP-5a is located around the NA site [63] and may be involved in inhibitor binding. The D-loop residue Tyr1203 is oriented towards the NA site in conformation I, and Phe1208 in conformation II (Figure S2). The phenyl ring of the D-loop residue occupies a similar position in conformations I and II, indicating that the D-loop can mediate nonpolar interactions with NA site ligands in both possible conformational states.

**Figure 2:**
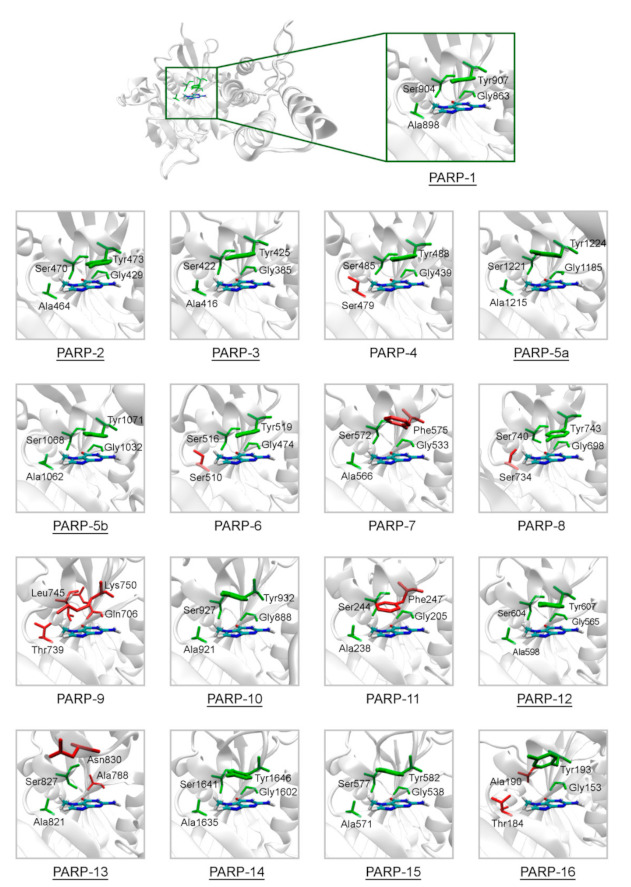
Structure of the NA binding site in PARPs 1–16. Underlined PARP structures (1–3, 5a, 5b, 10, 12–16) were obtained from the Protein Data Bank, and other structures (4, 6–9, 11) were obtained by homology modeling. Amino acid substitutions in the NA site are marked in red. The NA site architecture is quite similar in PARPs 1–3, 5a, 5b, 10, 12, 14, and 15 due to the lack of substitutions. The coordinates of the 7-MG molecule (colored by atom type) are transferred from the docking model of PARP-1–7-MG complex.

The representative crystal structures of PARPs shown in Figure 2 provide a good starting point for modeling intermolecular interactions with NA mimics and probing the selectivity. We have chosen 7-methylguanine (7-MG) as a probe PARP inhibitor for the following reasons. (i) 7-MG is a promising competitive inhibitor of the founding family member PARP-1 [64,65,66,67], (ii) 7-MG is a small NA mimic that occupies only the NA binding region, forming crucial interactions with the Gly863, Ala898, Ser904, and Tyr907 residues [51,65], (iii) high-quality force field parameters of 7-MG are available and ready for use in molecular modeling [64]. The 7-MG molecule was docked into the NA binding site of PARPs, and then its position was refined using MD simulation and analyzed.

**Figure 3:**
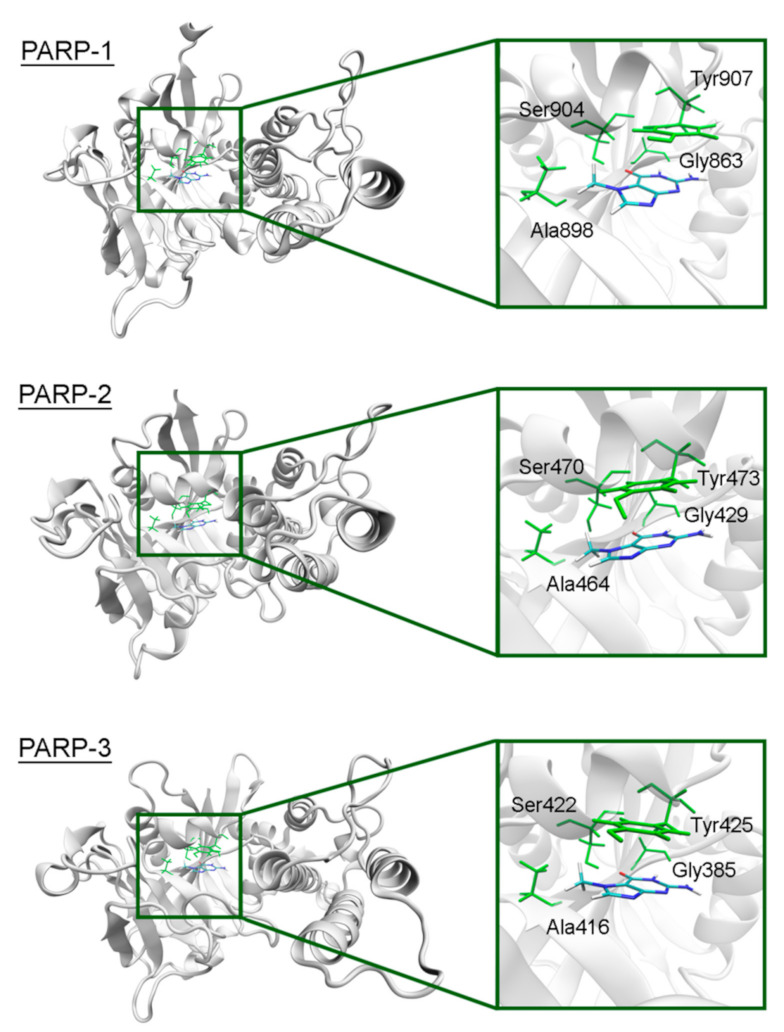
7-MG position in the NA binding site of PARPs 1–3 obtained using MD modeling. The NA site residues are colored in green.

The overall structure of the catalytic domain is similar in PARPs 1–3 and includes an (ADP-ribosyl) transferase subdomain and a regulatory helical subdomain [68]. The residues of the NA binding site are conserved in these proteins and form interactions with 7-MG typical for PARP inhibitors: Two hydrogen bonds between Gly863/429/385 (PARP-1/2/3) and the lactam group, which act simultaneously as a donor and an acceptor, a hydrogen bond between Ser904/470/422 and the lactam oxygen, hydrophobic contact between Ala898/464/416 and the 7-methyl group, *π*-stacking between Tyr907/473/425 and the 7-MG fused rings (Figure 3, Figure S3 showing a close-up view of the NA site, and Table S2 providing mean distances and angles from 10 ns simulation). It is, therefore, not surprising that 7-MG inhibits PARP-1 and 2 with comparable potency [64].

**Figure 4:**
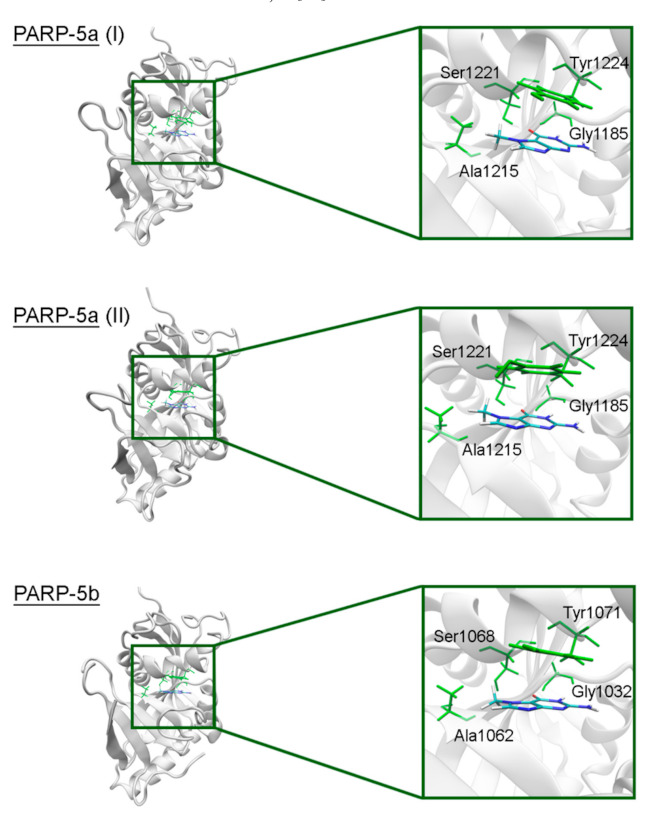
7-MG position in the NA binding site of PARP-5a (conformations I and II) and 5b obtained using MD modeling. The NA site residues are colored in green.

**Figure 5:**
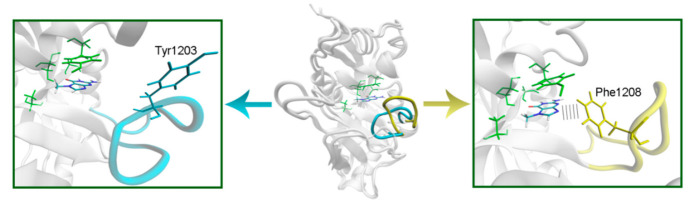
D-loop conformation in PARP-5a and its interaction with the NA binding site revealed by MD modeling. The NA site residues are colored in green, D-loop conformation I is shown in blue, conformation II in yellow.

In PARPs 5a and 5b, also known as tankyrases, the NA site residues are the same as in PARPs 1–3 and mediate similar interactions with 7-MG (Figure 4, Table S2), but additional intermolecular contacts may be formed due to the D-loop mobility. In the starting model of PARP-5a conformation I, the Tyr1203 side chain contacted with an aromatic nitrogen of 7-MG, but during the MD simulation, it moved away (without affecting the main chain coordinates). Meanwhile, the unfavorable contact with the Phe1208 side chain was persistent in conformation II (Figure 5). The starting D-loop position in the PARP-5b model was similar to conformation I of PARP-5a, and the corresponding Tyr1050 residue also moved away from 7-MG during the simulation. Both PARP-5a and 5b models indicate that D-loop interactions with donor/acceptor atoms of NA mimics are unfavorable, in accordance with our preliminary experimental data demonstrating the 7-MG selectivity for PARP-1 over PARP-5b (Table S3) [69]. However, more hydrophobic inhibitors may be selective against tankyrases due to additional contacts with Tyr1203/1050 or Phe1208. For example, the chloro substituent of an aromatic inhibitor interacts with the Tyr1050 side chain in the PARP-5b structure 4j1z [70].

**Figure 6:**
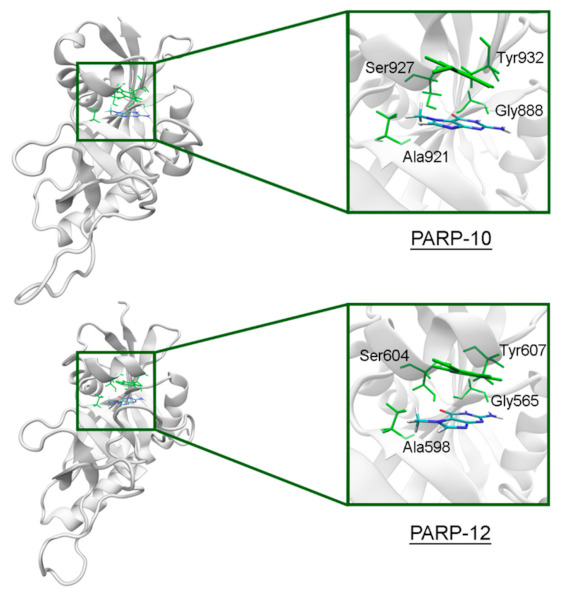
7-MG position in the NA binding site of PARP-10 and 12 obtained using MD modeling. The NA site residues are colored in green.

**Figure 7:**
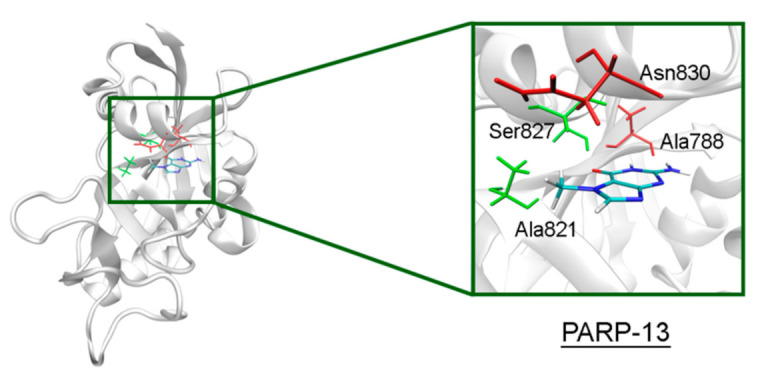
7-MG position in the NA binding site of PARP-13 obtained using MD modeling. The conserved NA site residues are colored in green, and unique residues in red.

In PARP-10 and 12, the NA site residues are also conserved (Figure 6). However, the hydrogen bond with the Ser604 residue in the active site of PARP-12 was characterized by an increased mean distance (Table S2) because its side chain periodically formed an alternative bond with the Asp600 main chain. In PARP-13 the crucial Gly residue is replaced with Ala788 whose methyl group is oriented away from the inhibitor and does not significantly affect the formation of hydrogen bonds between the main chain and lactam group. The Tyr residue is replaced with Asn830 in the NA site of PARP-13, which results in loss of the stacking interaction (Figure 7).

**Figure 8:**
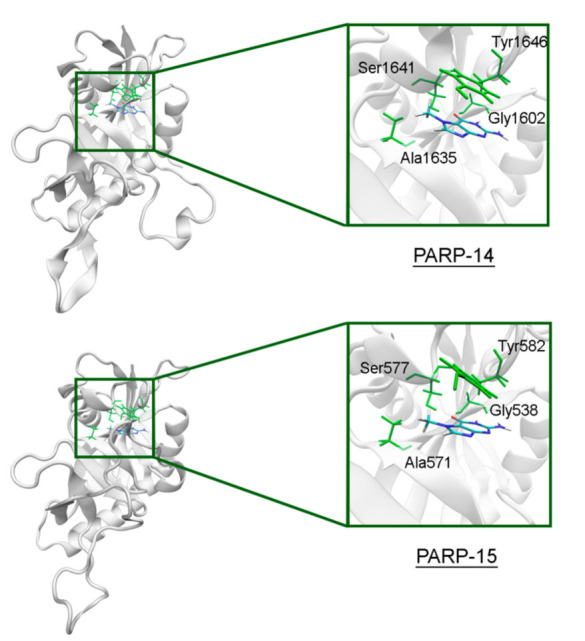
7-MG position in the NA binding site of PARP-14 and 15 obtained using MD modeling. The NA site residues are colored in green.

**Figure 9:**
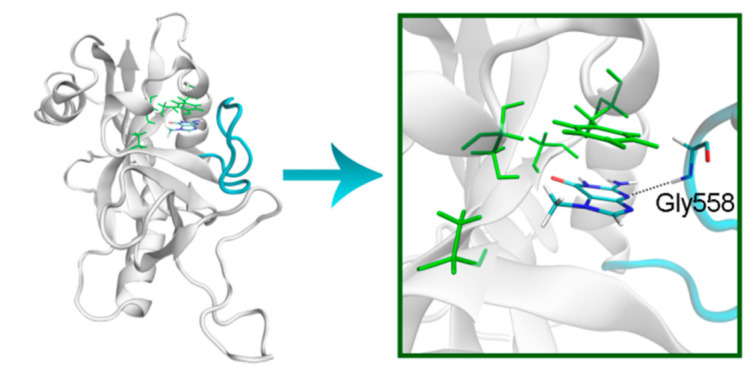
D-loop conformation in PARP-15 and its interaction with 7-MG. The NA binding site is colored in green, and D-loop in blue.

**Figure 10:**
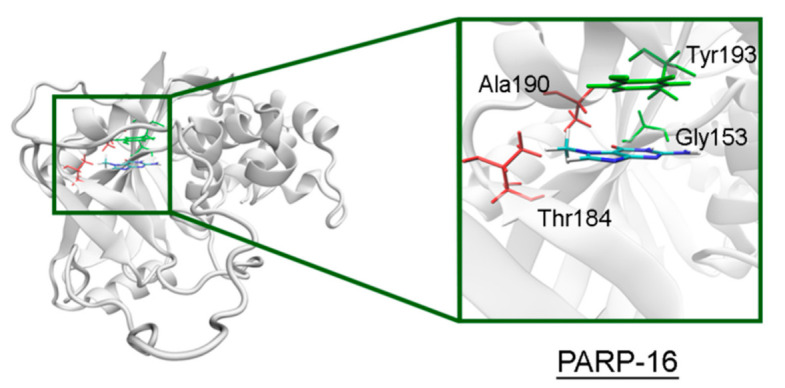
7-MG position in the NA binding site of PARP-16 obtained using MD modeling. The conserved NA site residues are colored in green, and unique residues in red. The C^γ^ atom of Thr184 forms a hydrophobic contact with the 7-MG methyl group.

The NA site residues in PARP-14 and 15 are the same as in PARPs 1–3 (Figure 8). However, during the simulation of PARP-15 an additional hydrogen bond was formed between the aromatic nitrogen of 7-MG and the D-loop residue Gly558 (Figure 9). In PARP-16 one hydrogen bond with the lactam group is lost due to the Ser replacement by Ala190, while the replacement of another NA site residue Ala by Thr184 does not affect the ability to form favorable contacts with the inhibitor’s hydrophobic group (Figure 10, Table S2).

### 2.3. Modeling of PARPs with Unknown Structures

**Figure 11:**
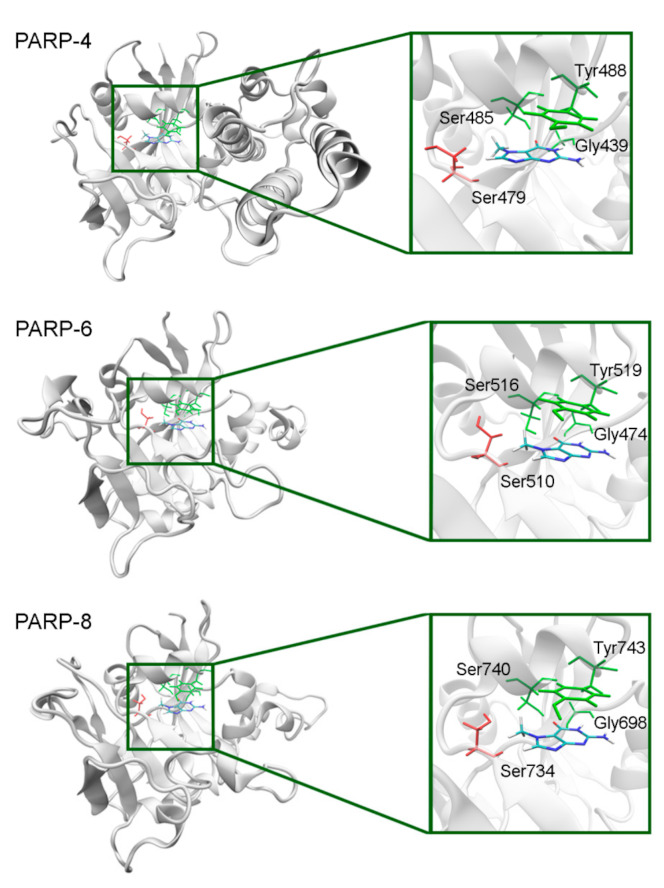
7-MG position in the NA binding site of PARPs 4, 6, and 8 obtained using homology modeling. The conserved NA site residues are colored in green and unique residues in red. The C^β^ atom of Ser479/510/734 forms a hydrophobic contact with the 7-MG methyl group.

The NA binding site architecture in PARPs of unknown structure (4, 6–9, and 11) was predicted and studied using homology modeling (template structures are listed in Table S4). We consider such an approach reasonable because the functional sites of the protein tend to be more conserved in evolution (than the rest of the fold) and thus more accurately modeled [71]. The coordinates of 7-MG were transferred into the NA site of the obtained PARP models and then optimized. The modeled fold of PARP-4 was similar to its homologs PARPs 1–3, and the replacement of Ala with Ser479 did not affect significantly the NA site structure because the Ser479 side chain was oriented away from the inhibitor (Figure 11, Table S5). Similarly to PARP-4, PARP-6, and PARP-8 contain non-essential substitutions of Ala by Ser510 and Ser734, respectively (Figure 11).

**Figure 12:**
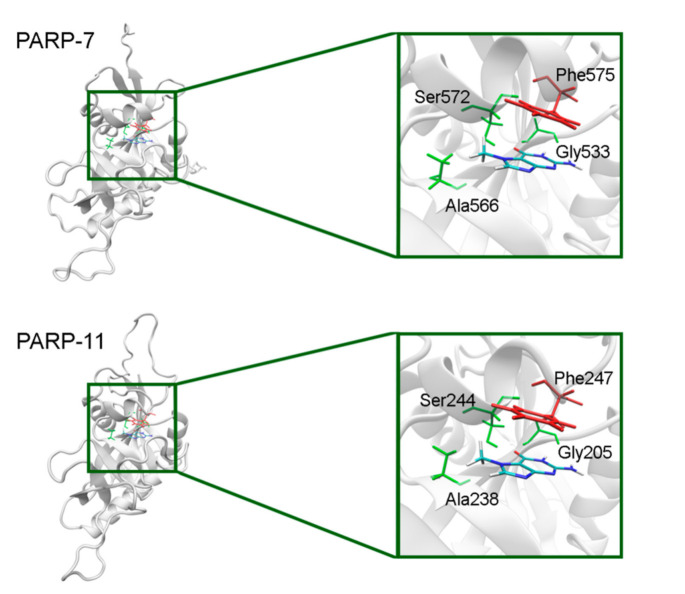
7-MG position in the NA binding site of PARP-7 and 11 obtained using homology modeling. The conserved NA site residues are colored in green and unique residues in red.

**Figure 13:**
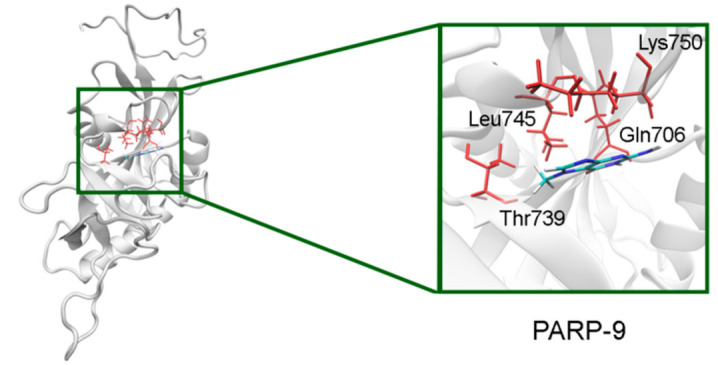
7-MG position in the NA binding site of PARP-9 obtained using homology modeling. The Gln706 main chain forms hydrogen bonds with the lactam group. The C^γ^ atom of Thr739 forms a hydrophobic contact with the 7-MG methyl group.

In PARP-7 and 11, the replacement of Tyr by Phe575/Phe247 does not disrupt *π*-stacking because this interaction with the inhibitor can be mediated by a phenyl ring of both Tyr and Phe residues (Figure 12, Table S5). Lastly, the modeled PARP-9 protein is a PARP family member in which all the NA site residues are substituted (Figure 13). Gly is replaced by Gln706 whose side chain is oriented away from the inhibitor and does not affect the formation of hydrogen bonds between the main chain and lactam group. Ser is replaced by Leu745, which results in the loss of an additional bond with the lactam group. Similarly to PARP-16, Ala is replaced by Thr739, which does not significantly affect the hydrophobic interaction. The stacking interaction with the inhibitor is lost in PARP-9 due to the replacement of Tyr with Lys750.

## 3. Discussion

PARP inhibitors represent a promising new component of cancer chemotherapy, and a lot of attention is given to the design of compounds selective towards distinct PARP family members. In particular, PARP-1 and 2 are involved in the elimination of single- and double-strand breaks of DNA, and tankyrases (PARP-5a and 5b) modify the axin protein in the Wnt pathway to promote cell proliferation. Therefore, PARP-1/2 inhibitors can be effective against BRCA-deficient tumors [72,73], whilst tankyrase inhibitors against tumors with abnormal Wnt signaling [74,75,76,77]. Here, we present a detailed study of the NA site, crucial for substrate and inhibitor binding, in PARP family proteins. Bioinformatic analysis and molecular modeling with a probe 7-MG inhibitor allowed us to identify structural features of the NA site in PARPs 1–16 important for the selective binding. It should be noted that a recently published paper by Kam et al., also dedicated to an in silico family-wide analysis of PARPs, is mostly focused on new potential targets for inhibition beyond the NA site [78], and therefore, our findings do not conflict with or duplicate previously reported data.

The most prevalent NA site residue at position 863 (PARP-1 numbering) is Gly which forms key hydrogen bonds with the inhibitor’s lactam group in the obtained PARP models. The replacement of Gly in PARP-9 and 13 (with Gln and Ala, respectively) does not significantly affect the formation of these main-chain hydrogen bonds. Ala, Ser, or Thr at position 898 provide favorable contacts with the inhibitor’s hydrophobic group. Ser at position 904 forms an additional hydrogen bond with the lactam group, except PARP-9 and 16 where it is replaced by Leu and Ala, respectively. Tyr or Phe at position 907 form *π*-stacking interactions, except PARP-9 and 13 containing substitutions with Lys and Asn, respectively. Thus, substitutions at positions 904 and 907 presumably provide selectivity of NA mimics for PARPs 1–8, 10–12, 14, and 15 over PARPs 9, 13, and 16. The mobility of the D-loop around the NA site of PARPs 5a, 5b and 15 can result in additional interactions: hydrophobic (PARP-5a, 5b) or polar (PARP-15). This may be exploited in the design of selective inhibitors of PARP-1/2 or PARP-5a/5b: more hydrophobic NA mimics would tend to bind with PARP-5a and 5b, while more polar compounds, forming unfavorable contacts with D-loop, would preferentially target PARP-1 and 2. As surgery remains the primary modality of cure in cancers associated with aberrant Wnt signaling, additional targeted treatments with selective PARP-5a/5b inhibitors may be of great interest [79,80].

## 4. Materials and Methods

Amino acid sequences of the catalytic domain of PARPs 1–16 were obtained from the UniProt database [81]. To construct a multiple alignment of PARPs, various state-of-the-art methods (COBALT, PROMALS3D, Matt, Clustal Omega) were independently used, followed by manual expert evaluation, and the alignment by Clustal Omega [82,83,84] was found to be the most accurate due to correct superimposition of key NA site residues.

Crystal structures of the catalytic domain of PARPs 1–3, 5a, 5b, 10, and 12–16 were obtained from the Protein Data Bank [85], superimposed with Matt 1.0 [86], and manually clustered based on the NA binding site conformation. Molecular models of PARPs 1–3, 5a (conformations I and II), 5b, 10, and 12–16 were then constructed based on the selected representative crystal structures. The coordinates of missing residues of the PARP catalytic domain were predicted with Modeller 9.20 or transferred from other structures (Table S6) [87,88]. Next, the protein structures were optimized with AmberTools 15 and Amber 14 [89,90], according to the following protocol. Hydrogen atoms were added to the structure considering ionization of amino acid residues, and then it was solvated by 12 Å-thick layer of TIP3P water. Chloride or sodium ions were added to neutralize the net charge. The energy minimization was performed with positional restraints on heavy atoms of the protein (2500 steepest descent steps + 2500 conjugate gradient steps). The 7-MG molecule was docked into the active site of the PARP models with Lead Finder 1.1.16 [91,92]. The obtained PARP–7-MG complexes were re-optimized in two stages, one with positional restraints on the protein and inhibitor atoms (2500 steepest descent steps + 2500 conjugate gradient steps) and the other without restraints (5000 steepest descent steps + 5000 conjugate gradient steps). The system was then heated up from 0 to 300 K (50 ps, constant volume) and equilibrated at 300 K (500 ps, constant pressure). Lastly, 10 ns trajectory of equilibrium simulation was calculated and analyzed. Structures of PARPs 4, 6–9, and 11 were obtained using homology modeling. Close homologs of these family members were identified with a HHpred server [93,94] and used as templates for Modeller 9.20. 7-MG coordinates were transferred from the docking model of PARP-1 complex, and the obtained homology models of PARP–7-MG complexes were then energy minimized in two stages, as described above.

Control data for energy minimization and MD simulation are provided in Table S7. The *ff14SB* force field [95] was used to describe the protein with molecular mechanics, and recently developed parameters [64] were used to describe the 7-MG molecule. VMD 1.9.2 was used for the visualization of structures [96].

## 5. Conclusions

The present paper systematically describes the architecture of the NA binding site in 17 PARP family proteins (PARPs 1–4, 5a, 5b, 6–16) and can serve as a useful guide to estimate the selectivity of NA mimics towards distinct family members. Certain factors may lead to the selective inhibition: (i) Mutations in the NA site and (ii) D-loop mobility around the NA site. An important finding of our study is that only in tankyrases (PARP-5a and 5b) the mobile D-loop can form additional hydrophobic contacts with NA mimics, which provides opportunities for the development of highly selective tankyrase inhibitors as promising anticancer agents.

## Figures and Tables

**Table 1:** Catalytic activity and cellular functions of PARP family proteins.

PARP	Activity	Function
**1**	poly ^1^	DNA repair enzymes regulating transcription and eliminating single- and double-strand breaks of DNA [13,14,15,16,17]
**2**	poly	
**3**	mono ^2^	DNA repair [18,19]
**4**	mono	poorly studied
**5a**	poly	tankyrase enzymes regulating the Wnt signaling pathway and controlling cellular proliferation and differentiation [20,21,22,23]
**5b**	poly	
**6**	mono	tumor suppressor regulating Survivin expression [24]
**7**	mono	poorly studied
**8**	mono	poorly studied
**9**	inactive	poorly studied
**10**	mono	regulates Aurora A and suppresses tumor metastasis [25]
**11**	mono	poorly studied
**12**	mono	suppresses Zika virus infection [26]
**13**	inactive	regulates the cellular response to stress [27]
**14**	mono	suppresses kinase-mediated apoptosis [28]
**15**	mono	poorly studied
**16**	mono	poorly studied

^1^ Poly(ADP-ribosyl)ation. ^2^ Mono(ADP-ribosyl)ation.

**Table 2:** Residues of the NA binding site in PARPs 1–16. Amino acid replacements are marked in gray.

PARP	Residues of the NA Site				PDB ID ^1^
**1**	Gly863	Ala898	Ser904	Tyr907	4zzz [54]
**2**	Gly429	Ala464	Ser470	Tyr473	4zzx [54]
**3**	Gly385	Ala416	Ser422	Tyr425	4gv2 [55]
**4**	Gly439	Ser479	Ser485	Tyr488	-
**5a**	Gly1185	Ala1215	Ser1221	Tyr1224	4w6e/4msg [56,57]
**5b**	Gly1032	Ala1062	Ser1068	Tyr1071	5nwg [58]
**6**	Gly474	Ser510	Ser516	Tyr519	-
**7**	Gly533	Ala566	Ser572	Phe575	-
**8**	Gly698	Ser734	Ser740	Tyr743	-
**9**	Gln706	Thr739	Leu745	Lys750	-
**10**	Gly888	Ala921	Ser927	Tyr932	5lx6 [59]
**11**	Gly205	Ala238	Ser244	Phe247	-
**12**	Gly565	Ala598	Ser604	Tyr607	2pqf [60]
**13**	Ala788	Ala821	Ser827	Asn830	2x5y [60]
**14**	Gly1602	Ala1635	Ser1641	Tyr1646	3smj [37]
**15**	Gly538	Ala571	Ser577	Tyr582	4f0e [61]
**16**	Gly153	Thr184	Ala190	Tyr193	4f0d [62]

^1^ Representative crystal structures.

## Data Availability

The data presented in this study are available on request from the corresponding author.

## Supplementary Materials

The following are available online at https://www.mdpi.com/2072-6694/13/6/1201/s1, Figure S1: Cluster of similar conformations of the NA binding site in PARP-1 crystal structures, Figure S2: Two possible conformations of the D-loop in crystal structures of PARP-5a, Figure S3: Interactions of 7-MG in the NA binding site of PARP-1 revealed by molecular modeling, Table S1: Crystal structures of PARPs used in the analysis of the NA binding site architecture, Table S2: Interactions between a probe inhibitor (7-MG) and NA site residues in PARPs revealed by 10-ns MD simulation, Table S3: Activity of PARP-1 and PARP-5b (tankyrase 2) at 7-MG concentration of 360 µM determined with an immunochemical assay, Table S4: PARPs of unknown structure and their close homologues, Table S5: Interactions between a probe inhibitor (7-MG) and NA site residues in PARPs revealed using homology modeling, Table S6: Missing residues in representative PARP structures, TableS7: Control data used for energy minimization and MD simulation of the PARP–7-MG complexes.

## Abbreviations

7-MG	7-methylguanine
MD	molecular dynamics
NA	nicotinamide
NAD^+^	nicotinamide adenine dinucleotide
PARP	poly(ADP-ribose)polymerase
