# Supplementary material for: Bioinformatic Analysis of the Nicotinamide Binding Site in Poly(ADP-Ribose) Polymerase Family Proteins
Source: Cancers (Basel). 2021 Mar 10;13(6):1201. doi: 10.3390/cancers13061201 (PMC8002165; doi:10.3390/cancers13061201)
Supplement: Supplementary file 1 [file cancers-13-01201-s001.pdf]

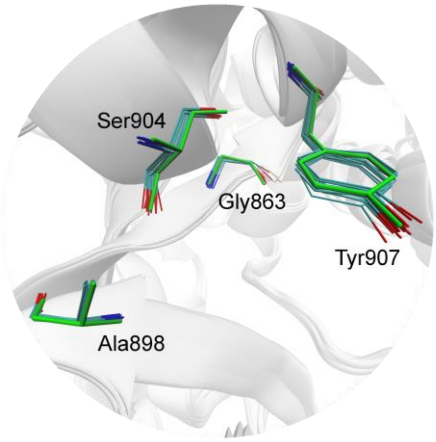

Figure S1. Cluster of similar conformations of the NA binding site in PARP-1 crystal structures. The selected representative structure of PARP-1 (PDB ID 4zzz, 1.9 Å resolution) is shown in green.

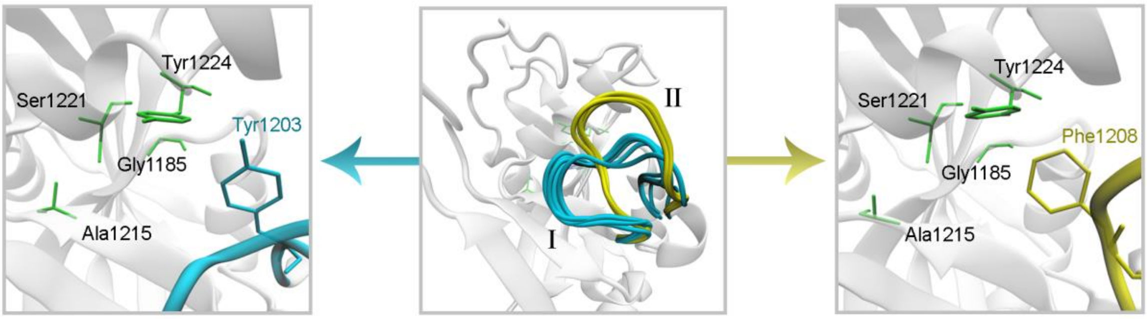

Figure S2. Two possible conformations of the D-loop in crystal structures of PARP-5a. Conformation I is shown in blue, conformation II in yellow.

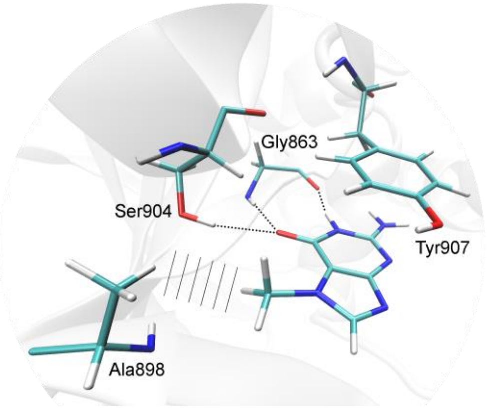

Figure S3. Interactions of 7-MG in the NA binding site of PARP-1 revealed by molecular modeling: hydrogen bonds with Gly863 and Ser904, hydrophobic contact with Ala898, and π-stacking with Tyr907.

Table S1. Crystal structures of PARPs used in the analysis of the NA binding site architecture. The selected representative structures are marked in gray, structures excluded as outliers/minor conformations are indicated with a strikethrough.

PARP	Available PDB Structures
**1**	1uk0, 1uk1, 1wok, ~~2n8a~~, 2rcw, 2rd6, 3gjw, 3gn7, 3l3l, 3l3m, ~~3od8~~, ~~3oda~~, ~~3odc~~, ~~3ode~~, 4av1, ~~4dqy~~, ~~4gv7~~, 4hhy, 4hhz, 4l6s, ~~4opx~~, ~~4oqa~~, ~~4oqb~~, 4pjt, 4r5w, 4r6e, 4rv6, 4und, 4uxb, 4xhu, 4zzz, 5a00, 5ds3, 5ha9, 5kpn, 5kpo, 5kpp, 5kpq, 5wrq, 5wry, 5wrz, 5ws0, 5ws1, 5wtc, 6bhv
**2**	3kcz, 3kjd, 4pjv, 4tvj, 4zzx, 4zzy, 5d5k, 5dsy
**3**	3c49, 3c4h, 3ce0, 3fhb, 4gv0, 4gv2, 4gv4, 4l6z, 4l70, 4l7l, 4l7n, 4l7o, 4l7p, 4l7r, 4l7u
**5a**	~~2rf5,~~ 3udd, 3uh2, 3uh4, ~~4dvi,~~ 4i9i, 4k4e, 4k4f, 4krs, 4li6, 4li7, 4li8, 4msg [1], 4msk, 4mt9, ~~4n3r~~, 4n4t, 4n4v, ~~4oa7~~, ~~4tos~~, ~~4tor~~, 4u6a, 4uuh, 4uw1, 4w5s, 4w6e [2], 5ebt, 5ece, 5ety
**5b**	3kr7, 3kr8, ~~3mhj~~, ~~3mhk~~, 3p0n, 3p0p, 3p0q, 3u9h, 3u9y, ~~3ua9~~, 3w51, 4avu, 4avw, ~~4bfp~~, ~~4bj9~~, ~~4bjb~~, ~~4bjc~~, 4bs4, 4bu3, 4bu5, 4bu6, 4bu7, 4bu8, 4bu9, 4bua, 4bud, 4bue, 4buf, 4bui, 4bus, 4but, 4buu, 4buv, 4buw, 4bux, 4buy, 4hki, 4hkk, 4hkn, 4hl5, 4hlf, 4hlg, 4hlh, 4hlk, 4hlm, 4hmh, ~~4hyf~~, 4iue, 4j1z, 4j21, 4j22, 4j3l, 4j3m, 4kzl, 4kzq, 4kzu, 4l09, 4l0b, 4l0i, 4l0s, 4l0t, 4l0v, 4l10, 4l2f, 4l2g, 4l2k, 4l31, 4l32, 4l33, 4l34, 4m7b, ~~4pml~~, ~~4pnl~~, ~~4pnm~~, ~~4pnn~~, ~~4pnq~~, ~~4pnr~~, ~~4pns~~, ~~4pnt~~, ~~4tju~~, ~~4tjw~~, ~~4tjy~~, ~~4tk0~~, ~~4tk5~~, ~~4tkf~~, ~~4tkg~~, ~~4tki,~~ 4ufu, 4ufy, 4uhg, 4ui3, 4ui4, 4ui5, 4ui6, 4ui7, 4ui8, 4uvl, 4uvn, 4uvo, 4uvp, 4uvs, 4uvt, 4uvu, 4uvv, 4uvw, 4uvx, 4uvy, 4uvz, 4ux4, 4w5i, ~~5adq~~, ~~5adr~~, ~~5ads~~, ~~5adt~~, ~~5aeh~~, 5aku, 5akw, 5al1, 5al2, 5al3, 5al4, 5al5, 5c5p, 5c5q, 5c5r, 5dcz, 5fpf, 5fpg, ~~5nob~~, 5nsp, 5nsx, 5nt0, 5nt4, 5nut, 5nvc, 5nve, 5nvf, 5nvh, 5nwd, 5nwg, 5nxe
**10**	3hkv, 5lx6
**12**	2pqf
**13**	2x5y, 4x52
**14**	~~3goy,~~ 3se2, ~~3smi~~, 3smj, 4f1l, 4f1q, 4py4, 5lxp, 5lyh, 5nqe, 5v7t, ~~5v7w~~
**15**	3blj, 3gey, 4f0e
**16**	4f0d

[1] D-loop conformation II, [2] D-loop conformation I.

Table S2. Interactions between a probe inhibitor (7-MG) and NA site residues in PARPs 1–3, 5a, 5b, 10, and 12–16 revealed by 10-ns MD simulation. Mean values are presented with standard deviations.

**PARP-1**		
	**Distance, Å**	
	7-MG:CO:O ··· Gly863:H	2.1 ± 0.2
	7-MG:CO:O ··· Ser904:OG:HG	1.9 ± 0.3
	7-MG:NH:H ··· Gly863:O	1.9 ± 0.1
	7-MG:CH_3:C ··· Ala898:CB	3.9 ± 0.3
	C(7-MG fused rings) ··· *C*(Tyr907 benzene ring) [1]	3.6 ± 0.2
	**Angle, deg**	
	7-MG Г:CO:O ··· Gly863:H ··· Gly863:N	160 ± 12
	7-MG:CO:O ··· Ser904:OG:HG ··· Ser904:OG	158 ± 14
	7-MГ: MG:N ··· 7-MG:NH:H ··· Gly863:O	154 ± 10
**PARP-2**		
	**Distance, Å**	
	7-MG:CO:O ··· Gly429:H	2.1 ± 0.3
	7-MG:CO:O ··· Ser470:OG:HG	1.9 ± 0.3
	7-MG:NH:H ··· Gly429:O	1.9 ± 0.1
	7-MG:CH3:C ··· Ala464:CB	3.9 ± 0.3
	C(7-MG fused rings) ··· C(Tyr473 benzene ring)	3.6 ± 0.2
	**Angle, deg**	
	7-MG:CO:O ··· Gly429:H ··· Gly429:N	158 ± 13
	7-MG:CO:O ··· Ser470:OG:HG ··· Ser470:OG	157 ± 16
	7-MG:NH:N ··· 7-MG:NH:H ··· Gly428:O	153 ± 10
**PARP-3**		
	**Distance, Å**	
	7-MG:CO:O ··· Gly385:H	2.2 ± 0.3
	7-MG:CO:O ··· Ser422:OG:HG	2.1 ± 0.7
	7-MG:NH:H ··· Gly385:O	1.9 ± 0.1
	7-MG:CH3:C ··· Ala416:CB	3.8 ± 0.3
	C(7-MG fused rings) ··· C(Tyr425 benzene ring)	3.6 ± 0.2
	**Angle, deg**	
	7-MG:CO:O ··· Gly385:H ··· Gly385:N	148 ± 17
	7-MG:CO:O ··· Ser422:OG:HG ··· Ser422:OG	158 ± 25
	7-MG:NH:N ··· 7-MG:NH:H ··· Gly385:O	153 ± 11
**PARP-5a, D-loop conformation I**		
	**Distance, Å**	
	7-MG:CO:O ··· Gly1185:H	2.2 ± 0.2
	7-MG:CO:O ··· Ser1221:OG:HG	2.3 ± 1.0
	7-MG:NH:H ··· Gly1185:O	1.9 ± 0.1
	7-MG:CH3:C ··· Ala1215:CB	3.9 ± 0.3
	C(7-MG fused rings) ··· C(Tyr1224 benzene ring)	3.6 ± 0.2
	**Angle, deg**	
	7-MG:CO:O ··· Gly1185:H ··· Gly1185:N	161 ± 11
	7-MG:CO:O ··· Ser1221:OG:HG ··· Ser1221:OG	148 ± 33
	7-MG:NH:N ··· 7-MG:NH:H ··· Gly1185:O	148 ± 12
**PARP-5a, D-loop conformation II**		
	**Distance, Å**	
	7-MG:CO:O ··· Gly1185:H	2.1 ± 0.2
	7-MG:CO:O ··· Ser1221:OG:HG	2.1 ± 0.4
	7-MG:NH:H ··· Gly1185:O	1.9 ± 0.1
	7-MG:CH3:C ··· Ala1215:CB	3.7 ± 0.2
	C(7-MG fused rings) ··· C(Tyr1224 benzene ring)	3.7 ± 0.3
	**Angle, deg**	
	7-MG:CO:O ··· Gly1185:H ··· Gly1185:N	159 ± 12
	7-MG:CO:O ··· Ser1221:OG:HG ··· Ser1221:OG	154 ± 20
	7-MG:NH:N ··· 7-MG:NH:H ··· Gly1185:O	150 ± 12
**PARP-5b**		

	**Distance, Å**	
	7-MG:CO:O ··· Gly1032:H	2.1 ± 0.2
	7-MG:CO:O ··· Ser1068:OG:HG	2.3 ± 0.5
	7-MG:NH:H ··· Gly1032:O	1.9 ± 0.1
	7-MG:CH3:C ··· Ala1062:CB	3.8 ± 0.3
	C(7-MG fused rings) ··· C(Tyr1071 benzene ring)	3.6 ± 0.2
	**Angle, deg**	
	7-MG:CO:O ··· Gly1032:H ··· Gly1032:N	155 ± 13
	7-MG:CO:O ··· Ser1068:OG:HG ··· Ser1068:OG	148 ± 24
	7-MG:NH:N ··· 7-MG:NH:H ··· Gly1032:O	149 ± 12
**PARP-10**		
	**Distance, Å**	
	7-MG:CO:O ··· Gly888:H	1.9 ± 0.1
	7-MG:CO:O ··· Ser927:OG:HG	2.0 ± 0.3
	7-MG:NH:H ··· Gly888:O	2.1 ± 0.2
	7-MG:CH3:C ··· Ala921:CB	4.2 ± 0.4
	C(7-MG fused rings) ··· C(Tyr932 benzene ring)	4.0 ± 0.,3
	**Angle, deg**	
	7-MG:CO:O ··· Gly888:H ··· Gly888:N	162 ± 9
	7-MG:CO:O ··· Ser927:OG:HG ··· Ser927:OG	160 ± 13
	7-MG:NH:N ··· 7-MG:NH:H ··· Gly888:O	146 ± 10
**PARP-12**		
	**Distance, Å**	
	7-MG:CO:O ··· Gly565:H	1.9 ± 0.2
	7-MG:CO:O ··· Ser604:OG:HG	3.2 ± 1.5
	7-MG:NH:H ··· Gly565:O	2.1 ± 0.2
	7-MG:CH3:C ··· Ala598:CB	4.1 ± 0.4
	C(7-MG fused rings) ··· C(Tyr607 benzene ring)	3.7 ± 0.2
	**Angle, deg**	
	7-MG:CO:O ··· Gly565:H ··· Gly565:N	160 ± 11
	7-MG:CO:O ··· Ser604:OG:HG ··· Ser604:OG	111 ± 62
	7-MG:NH:N ··· 7-MG:NH:H ··· Gly565:O	147 ± 12
**PARP-13**		
	**Distance, Å**	
	7-MG:CO:O ··· Ala788:H	2.6 ± 0.5
	7-MG:CO:O ··· Ser827:OG:HG	1.9 ± 0.5
	7-MG:NH:H ··· Ala788:O	2.0 ± 0.2
	7-MG:CH3:C ··· Ala821:CB	3.9 ± 0.3
	**Angle, deg**	
	7-MG:CO:O ··· Ala788:H ··· Ala788:N	148 ± 16
	7-MG:CO:O ··· Ser827:OG:HG ··· Ser827:OG	158 ± 22
	7-MG:NH:N ··· 7-MG:NH:H ··· Ala788:O	149 ± 10
**PARP-14**		
	**Distance, Å**	
	7-MG:CO:O ··· Gly1602:H	1.9 ± 0.1
	7-MG:CO:O ··· Ser1641:OG:HG	2.0 ± 0.4
	7-MG:NH:H ··· Gly1602:O	2.6 ± 0.6
	7-MG:CH3:C ··· Ala1635:CB	4.1 ± 0.4
	C(7-MG fused rings) ··· C(Tyr1646 benzene ring)	4.0 ± 0.4
	**Angle, deg**	
	7-MG:CO:O ··· Gly1602:H ··· Gly1602:N	163 ± 10
	7-MG:CO:O ··· Ser1641:OG:HG ··· Ser1641:OG	160 ± 17
	7-MG:NH:N ··· 7-MG:NH:H ··· Gly1602:O	150 ± 12
**PARP-15**		
	**Distance, Å**	
	7-MG:CO:O ··· Gly538:H	1.9 ± 0.1
	7-MG:CO:O ··· Ser577:OG:HG	2.4 ± 1.2
	7-MG:NH:H ··· Gly538:O	2.1 ± 0.2

7-MG:CH3:C ··· Ala571:CB	4.7 ± 0.5
C(7-MG fused rings) ··· C(Tyr582 benzene ring)	4.5 ± 0.4
**Angle, deg**	
7-MG:CO:O ··· Gly538:H ··· Gly538:N	158 ± 11
7-MG:CO:O ··· Ser577:OG:HG ··· Ser577:OG	141 ± 45
7-MG:NH:N ··· 7-MG:NH:H ··· Gly538:O	150 ± 11
**PARP-16**	
**Distance, Å**	
7-MG:CO:O ··· Gly153:H	2.0 ± 0.2
7-MG:NH:H ··· Gly153:O	1.9 ± 0.1
7-MG:CH3:C ··· Thr184:CG2	3.8 ± 0.3
C(7-MG fused rings) ··· C(Tyr193 benzene ring)	3.6 ± 0.2
**Angle, deg**	
7-MG:CO:O ··· Gly153:H ··· Gly153:N	163 ± 9
7-MG:NH:N ··· 7-MG:NH:H ··· Gly153:O	151 ± 10

[1] Distance between the geometric center of 7-MG fused rings and the center of the Tyr benzene ring.

Table S3. Activity of PARP-1 and PARP-5b (tankyrase 2) at 7-MG concentration of 360 μM determined with an immunochemical assay, as described previously (Berishvili, V.P.; Kuimov, A.N.; et al. *Molecules*. **2020,** *25*, 3171). Mean values are presented with standard deviations.

PARP	Residual Activity %
**1**	14 ± 1
**5b**	54 ± 5

Table S4. PARPs of unknown structure and their close homologues.

PARP	Homologues	PDB Templates [1]
**4**	PARPs 1, 2, 3	4zzz, 4zzx, 4l7n
**6**	PARPs 1, 2, 3, 16	4zzz, 4zzx, 4l7n, 6hxs
**7**	PARPs 10, 12,14	3hkv, 2pqf, 3smj
**8**	PARPs 1, 2, 3, 16	4zzz, 4zzx, 4l7n, 6hxs
**9**	PARPs 10, 12, 15	3hkv, 2pqf, 6ek3
**11**	PARPs 10, 12, 14, 15	3hkv, 2pqf, 3smj, 6ek3

[1] Template structures used in homology modeling.

Table S5. Interactions between a probe inhibitor (7-MG) and NA site residues in PARPs 4, 6–9, and 11 revealed using homology modeling.

**PARP-4**	
**Distance, Å**	
7-MG:CO:O ··· Gly439:H	1.9
7-MG:CO:O ··· Ser485:OG:HG	1.8
7-MG:NH:H ··· Gly439:O	1.8
7-MG:CH3:C ··· Ser479:CB	3.6
C(7-MG fused rings) ··· C(Tyr488 benzene ring)	3.9
**PARP-6**	
**Distance, Å**	
7-MG:CO:O ··· Gly474:H	2.0
7-MG:CO:O ··· Ser516:OG:HG	1.8
7-MG:NH:H ··· Gly474:O	1.8
7-MG:CH3:C ··· Ser510:CB	3.5
C(7-MG fused rings) ··· C(Tyr519 benzene ring)	3.4
**PARP-7**	
**Distance, Å**	
7-MG:CO:O ··· Gly533:H	1.9
7-MG:CO:O ··· Ser572:OG:HG	1.8

7-MG:NH:H ··· Gly533:O	1.9
7-MG:CH3:C ··· Ala566:CB	3.8
C(7-MG fused rings) ··· C(Phe575 benzene ring)	3.5
**PARP-8**	
	**Distance, Å**
7-MG:CO:O ··· Gly698:H	2.0
7-MG:CO:O ··· Ser740:OG:HG	1.8
7-MG:NH:H ··· Gly698:O	1.8
7-MG:CH3:C ··· Ser734:CB	3.5
C(7-MG fused rings) ··· C(Tyr743 benzene ring)	3.4
**PARP-9**	
	**Distance, Å**
7-MG:CO:O ··· Gln706:H	1.9
7-MG:NH:H ··· Gln706:O	1.8
7-MG:CH3:C ··· Thr739:CB	4.2
**PARP-11**	
	**Distance, Å**
7-MG:CO:O ··· Gly205:H	1.9
7-MG:CO:O ··· Ser244:OG:HG	1.8
7-MG:NH:H ··· Gly205:O	1.9
7-MG:CH3:C ··· Ala238:CB	3.7
C(7-MG fused rings) ··· C(Phe247 benzene ring)	3.6

[1] Distance between the geometric center of 7-MG fused rings and the center of the Tyr/Phe benzene ring.

Table S6. Missing residues in representative PARP structures, whose coordinates were predicted or transferred from other PDB structures.

PARP	Representative Structure	Missing Residues
**1**	4zzz	784–786 (transferred from 5ws1)
**2**	4zzx	549, 550 (transferred from 3kjd)
**3**	4gv2	242–244 (transferred from 3c4h)
**5a**	4w6e [1]	1285 (transferred from 4uw1)
**12**	2pqf	644, 645 (predicted)
**16**	4f0d	171, 172, 223–252 (predicted)

[1] D-loop conformation I.

Table S7. Control data used for energy minimization and MD simulation of the PARP–7-MG complexes. For more information see the Amber Users' Manual.

**Energy Minimization, Stage 1**	**Energy Minimization, Stage 2**
&cntrl<br>imin=1, maxcyc=5000, ncyc=2500,<br>cut=10.0, ntb=1,<br>ntc=1, ntf=1,<br>ntpr=10,<br>ntr=1,<br>restraintmask=':1-354 & !@H=',<br>restraint_wt=2.0<br>/	&cntrl<br>imin=1, maxcyc=10000, ncyc=5000,<br>cut=10.0, ntb=1,<br>ntc=1, ntf=1,<br>ntpr=10<br>/
**MD simulation, heating**	**MD simulation, equilibration**
&cntrl<br>imin=0, irest=0, ntx=1,<br>nstlim=25000, dt=0.002,<br>ntc=2, ntf=2,<br>cut=10.0, ntb=1,<br>ntpr=500, ntwx=500,<br>ntt=3, gamma_ln=2.0,<br>tempi=0.0, temp0=300.0,<br>ntr=1, restraintmask=':1-354',<br>restraint_wt=1.0,<br>nmropt=1<br>/<br>&wt TYPE='TEMP0',<br>istep1=0, istep2=25000,<br>value1=0.1, value2=300.0 /<br>&wt TYPE='END' /	&cntrl<br>imin=0, irest=1, ntx=5,<br>nstlim=250000,<br>(to run 10 ns simulation, set<br>nstlim=5000000)<br>dt=0.002,<br>ntc=2, ntf=2,<br>cut=10.0, ntb=2, ntp=1, taup=2.0,<br>ntpr=1000, ntwx=1000, ntwr=50000,<br>ntt=3, gamma_ln=2.0,<br>temp0=300.0<br>/
